# Predictive factors for the Nursing Diagnoses in people living with Acquired Immune Deficiency Syndrome^1^

**DOI:** 10.1590/1518-8345.1103.2712

**Published:** 2016-07-04

**Authors:** Richardson Augusto Rosendo da Silva, Romanniny Hévillyn Silva Costa, Ana Raquel Cortês Nelson, Fernando Hiago da Silva Duarte, Nanete Caroline da Costa Prado, Eduardo Henrique Fagundes Rodrigues

**Affiliations:** 2 PhD, Adjunct Professor, Universidade Federal do Rio Grande do Norte, Natal, RN, Brazil.; 3 MSc, RN, Instituto Federal do Rio Grande do Norte, Natal, RN, Brazil.; 4 RN, Specialist in Occupational Health Nursing.; 5 RN, Student of the Specialization Course in Intensive Care Unit, Faculdade Metropolitana, CENPEX, Natal, RN, Brazil.; 6 RN, Student of the Specialization Multiprossional Course in Neonatal Intensive Care Unit, Universidade Federal do Rio Grande do Norte, Natal, RN, Brazil.; 7 Undergraduate student in Medicine, Departamento de Medicina, Universidade Federal do Rio Grande do Norte, Natal, RN, Brazil.

**Keywords:** Nursing Process, Nursing Diagnosis, Acquired Immunodeficiency Syndrome

## Abstract

**Objective:**

to identify the predictive factors for the nursing diagnoses in people living with Acquired Immune Deficiency Syndrome.

**Method:**

a cross-sectional study, undertaken with 113 people living with AIDS. The data were collected using an interview script and physical examination. Logistic regression was used for the data analysis, considering a level of significance of 10%.

**Results:**

the predictive factors identified were: for the nursing diagnosis of knowledge deficit-inadequate following of instructions and verbalization of the problem; for the nursing diagnosis of failure to adhere - years of study, behavior indicative of failure to adhere, participation in the treatment and forgetfulness; for the nursing diagnosis of sexual dysfunction - family income, reduced frequency of sexual practice, perceived deficit in sexual desire, perceived limitations imposed by the disease and altered body function.

**Conclusion:**

the predictive factors for these nursing diagnoses involved sociodemographic and clinical characteristics, defining characteristics, and related factors, which must be taken into consideration during the assistance provided by the nurse.

## Introduction

It is estimated that 35 million people are living with Human Immunodeficiency Virus (HIV). From the beginning of the AIDS epidemic (1980) through to December 2013, approximately 1.5 million deaths were identified which had Acquired Immune Deficiency Syndrome (AIDS) as their basic cause^1^. In this regard, AIDS remains a public health problem, demanding, therefore, attention both from managers and health professionals regarding measures for prevention, treatment, and the rehabilitation of the individuals. 

The nurse is inserted in this context as an important facilitator of the care for people living with AIDS, whether this involves undertaking the nursing procedures during inpatient treatment or assessing and providing guidance in relation to their state of health, tests, medications, diet or prevention of the transmission of the virus, among other issues. For this, it is necessary for these actions to be undertaken systematically. 

The Systematization of Nursing Care can take place based on the application of the Nursing Process, which is made up of five interrelated, interdependent and recurrent stages: data collection (the patient history), the nursing diagnosis, planning, implementation, and nursing evaluation. Through this, the nurse's practice is based on clinical judgment, greater organization, and quality of care. For this, it is essential for the professional to involve the people living with AIDS and their family members^2^.

In this respect, the identification of the nursing diagnoses and of their predictive factors, which are related to the human responses, facilitates the implementation of the nursing interventions which will be most appropriate for the real necessities of the people living with AIDS - and for their socioeconomic and cultural context. 

In order to support and justify the undertaking of this study, an integrative review was undertaken, looking for scientific productions published in the last five years on the issue in question. For this, the following databases provided through the Virtual Health Library (*Biblioteca Virtual em Saúde* - BVS) were used: Latin American and Caribbean Center on Health Sciences Information (LILACS) and Medical Literature Analysis and Retrieval System Online(MEDLINE); and Scopus and CINAHL (Cumulative Index to Nursing and Allied Health Literature),using the following combinations: Nursing diagnosis and Acquired Immune Deficiency Syndrome, Nursing process and Acquired Immune Deficiency Syndrome. It was ascertained that there were few studies addressing Nursing Diagnoses in people living with AIDS^2^ ^-^ ^6^, with studies being found on the profile and prevalence of the diagnoses, but an absence of investigations on their predictive factors. 

Based on the gap in knowledge identified on this issue, in the scientific literature, the question is raised: What are the predictive factors for the nursing diagnoses in people living with Acquired Immune Deficiency Syndrome? 

In the light of this, this study's objective was to identify the predictive factors for the nursing diagnoses in people living with Acquired Immune Deficiency Syndrome. 

## Method

A cross-sectional study, carried out in a public hospital located in the Northeast of Brazil. The study population consisted of 158 people diagnosed with AIDS receiving inpatient treatment in the hospital selected. For this, one episode of inpatient treatment was counted for each patient. The sample size was calculated based on the formula for finite populations, considering a level of confidence of 95% (Z∞=1.96), sampling error of 5% and the size of the population^7^. The sample was made up of 113 people living with AIDS, selected by convenience, and was of the consecutive type. 

The study included people diagnosed with AIDS, who were over 18 years old, and who had been receiving inpatient treatment for at least 12 hours. The study excluded those who did not have the physical or psychic conditions to participate in the study, as well as those who were not aware of the diagnosis of the disease. It is emphasized that people living with AIDS who were receiving inpatient treatment on a subsequent occasion, who had already been interviewed in the present study, were not included again in the sample. 

The collection took place in the period March - September 2014, corresponding to the time period necessary to reach the sample calculated. In approaching the subjects, the research proposal was presented, and the Terms of Free and Informed Consent were read. For this, the patients were given the time necessary for taking their decisions. If they wished to contribute to the study, a patient history was taken and the physical examination was undertaken in a separate room, in the above-mentioned hospital, respecting the patient's privacy. Throughout the data collection process, accessible language was used such that there would be no doubts or embarrassments.

The instruments used in collecting the data, for taking the patient history and carrying out the physical examination were adapted^2^ ^,^ ^8^ ^-^ ^9^, containing open and closed questions on the sociodemographic and clinical data. In addition to this, the researchers addressed the defining characteristics (signs and symptoms), and related factors/risk factors, subdivided into 12 domains (health promotion, nutrition, elimination and exchange, activity/rest, perception/cognition, self-perception, roles and relationships, sexuality, coping/tolerance regarding stress, safety/protection and comfort) present in the NANDA International Taxonomy II. For the adaptation, questions were added which were specific for people living with AIDS, such as: form of transmission, time since diagnosis, presence of opportunistic infections and co-infections, medications used and information on the laboratory tests undertaken. 

The instruments were validated by eight professors with Ph.Ds with experience in nursing diagnoses and Acquired Immune Deficiency Syndrome, who participated as judges. The evaluation of the instrument took place based on the classification of each item in relation to the judges' opinion regarding agreement or disagreement relating to the item remaining in the instruments. Besides this, suggestions could also be made. Items which reached a rate of agreement of ≥0.80 among the professors who participated in the study were incorporated into the instruments, being considered validated. The above-mentioned instruments were applied, in the form of a pretest, to 10 people living with AIDS, in the study locale. There was no need for changes, and the participants were included in the study's sample. 

The NANDA International, 2012-2014 version^9^ was used to classify the nursing diagnoses, the defining characteristics, and related factors or risk factors. The process of diagnostic inference was undertaken by two researchers and took place in two stages: analysis (categorization of the data and identification of the gaps) and synthesis (grouping, comparison, identification, and relating of the causative factors).

A database was constructed which recorded all of the sociodemographic and clinical variables, the defining characteristics, the related factors and the nursing diagnoses identified. The analysis of the data was undertaken descriptively, through obtaining absolute and relative frequencies. For the numerical variables, the measurements of central tendency were presented. 

Inferential statistics were calculated through logistic regression using the stepwise method in order to identify the predictors of the nursing diagnoses which influenced the process of establishing the human responses, presented by people living with AIDS; a level of significance of 10% was considered. It is emphasized that it was impossible to undertake statistical tests for the nursing diagnosis of "ineffective protection", as this presented a frequency of 100% among the participants in the present study. 

As a result, the logistic regression was undertaken for the three diagnoses most prevalent in this study, with the sociodemographic and clinical variables, contained in the research instrument (sex, age group, marital situation, origin, religious belief, years in education, family income, occupational situation, current co-infection with HIV, current opportunistic infection, existence of other diseases, use of antiretrovirals, adverse reactions to the antiretrovirals in the first weeks, reason for the current episode of inpatient treatment, abandonment of treatment, difficulty in accessing the service, smoker, user of alcohol and illicit drugs, difficulty in learning new things, existence of doubt regarding the treatment, satisfaction with appearance and lifestyle, body alteration related to the disease, active sexual life, and reduced frequency of sexual practice), as well as the defining characteristics and related factors, which presented a prevalence of 15%. 

The research project was approved by the Committee for Ethics in Research with Human Beings, of the Federal University of Rio Grande do Norte (Opinion N. 508.445/2014), respecting the national and international rules for ethics in research involving human beings. 

## Results

The majority of people living with AIDS are male (n=82/72.6%), with a mean age of 39 years old (±9.81), are heterosexual (n=81/71.7%), have studied for up to eight years (n=74/65.5%), have a family income of up to one minimum salary (n=66/58.5%), and live in the interior - that is, the rural areas - of the State (n=60/67.8%). On average, the time since diagnosis with AIDS was 5 years (±5.38), and 69 (n=78) had already abandoned treatment through lack of belief (n=16/20.5%).

A total of 56 nursing diagnoses was identified, with four being prevalent in more than 50% of the people living with AIDS, these being: ineffective protection (n=113/100%), knowledge deficit (n=91/80.5%), failure to adhere (n=78/69%) and sexual dysfunction (n=61/54%).The frequencies of the defining characteristics of the Nursing Diagnosis (ND) of ineffective protection were: lack of immunity (n=113/100%), change in coagulation (n=76/67.2%), fatigue (n=70/61.9%), weakness (n=52/46%), and dyspnea (n=28/24.7%). On the other hand, for the related factors, those prevalent were: immunological disorders (n=113/100%), abnormal blood profiles (n=96/84.9%), drug abuse (n=51/45.1%), inadequate nutrition (n=25/22.1%) and drug therapy (n=16/14.1%). Table 1 presents the distribution of the predictive factors for the Nursing Diagnoses identified in patients with Acquired Immune Deficiency Syndrome, apart from the ND of ineffective protection.

**Table 1:** Distribution of the predictive factors for the Nursing Diagnoses identified in patients with Acquired Immune Deficiency Syndrome. Natal, State of Rio Grande do Norte (RN), Brazil, 2014

Predictive factors		Nursing Diagnoses			P value	Cox & Snell R^2^	NagelkerkeR^2^
		Present (%)	Absent (%)				
Inadequate following of instructions		Knowledge deficit					
	Present	80.5	3.5		0.002	0.627	1.000
	Absent	0.0	16.0				
Verbalization of the problem							
	Present	80.5	5.5		0.002		
	Absent	0.0	14.0				
Years of study		Failure to adhere					
	Up to 8 years of study	44.2	21.3		0.071		
	Over 8 years of study	25.3	9.2				
Participation in the treatment							
	Present	59.9	6.4		0.076		
	Absent	5.0	28.7			0.705	1.000
Behavior indicative of a lack of adherence							
	Present	61.9	4.4		0.005		
	Absent	7.0	26.7				
Forgetfulness							
	Present	64.6	0.0		0.097		
	Absent	4.4	31.0				
Family income		Sexual dysfunction					
	Up to 1 minimum salary	31.8	26.7		0.098		
	Over 1 minimum salary	21.9	19.6				
Reduced frequency of sexual practice							
	Present	53.9	2.6		0.038		
	Absent	0	43.5				
Perceived Deficitin sexual desire						0.749	1.000
	Present	52.2	8.8		0.005		
	Absent	1.8	37.2				
Perceived limitations imposed by the disease							
	Present	51.3	7.9		0.005		
	Absent	2.6	38.2				
Altered body function							
	Present	43.4	10.6		0.058		
	Absent	10.6	35.4				

The predictive factors identified were: for the nursing diagnosis of knowledge deficit - inadequate following of instructions, and verbalization of the problem; for the nursing diagnosis of failure to adhere - years of study, behavior indicative of a lack of adherence, participation in the treatment and forgetfulness, and for the nursing diagnosis of sexual dysfunction - family income, reduced frequency of sexual practice, perceived deficit in sexual desire, perceived limitations imposed by the disease, and altered body function.

## Discussion

The AIDS epidemic, in Brazil, in recent years, has been marked by a change in the epidemiological profile, marked by heterosexualization, povertization and interiorization (that is, its spread from cities into rural areas)^10^.Studies on people living with AIDS have been undertaken predominantly with male patients aged up to 40 years old, resembling that found in this study^4^ ^-^ ^6^.

The nursing diagnosis of ineffective protection presented, in general, defining characteristics and related factors linked to the change in immunological status, including the changes of the blood cells. Studies undertaken previously corroborate this finding^2^ ^,^ ^4^ ^-^ ^5^.

It is known that HIV works by linking itself to the surface of the individual's immunological cells, mainly destroying the CD4 cells, although it can also reach blood cells, platelets and leukocytes, leading to a situation of fatigue, dyspnea, anemia, infections and hemorrhage. These hematological changes can also be somatized by the immunosuppressive effect itself of the opportunistic infections and neoplastic infections and by the myelotoxic effects which some antiretroviral medications, or corticosteroid and antineoplastic medications, can cause^11^.

The literature also points to the presence of gastrointestinal changes as a predictive factor for the worsening of the immunological situation, as diarrhea, in conjunction with the action itself of the virus, provokes change in the absorption of nutrients and the metabolism of lipids, contributing to the appearance of a situation of malnutrition^12^ ^-^ ^14^.

Among the opportunistic infections and co-infections, emphasis is placed upon: pneumocystis, toxoplasmosis, tuberculosis, cryptococcal meningitis and infection by citomegalovirus and herpes zoster, among others^13^ ^,^ ^15^.

Some of these infections are not related exclusively to the reduction in the CD4 T cells, as in the cases of tuberculosis and candidiasis. The paradoxal reaction, resulting from the treatment of tuberculosis, can be caused by the liberation of the protein from the bacillus during its destruction. This protein content may be understood by the organism as an anti-gen, thus causing an exacerbated innate and adaptive immune response. Infection by *Candida* is observed with any level of CD4 T cells, such that the reduction in these cells influences only its frequency and severity^16^.

In the light of the above, the appearance of the disease's complications can be related to various factors, namely: mechanisms of the action of the virus, paradoxal effect of the treatment of tuberculosis, changes in the leukocyte cells and effects of the antiretroviral medications. These factors can lead to absence of interest in the treatment, contributing to its abandonment. 

The nurse needs to monitor the clinical situation of the people living with AIDS, being attentive to the blood markers and symptomatology, with an aim to identifying the complications caused by the disease. Furthermore, it is important for these people to be aware regarding their current clinical status and prognosis and, above all, regarding how they can contribute to minimizing these. 

Regarding the nursing diagnosis of knowledge deficit, it is known that the inadequate following of instructions, and verbalization of the problem, can influence the way that people living with AIDS deal with the disease, as well as their adherence to the treatment and to healthy behaviors^17^.

In this regard, the nurse must encourage people living with AIDS to reports their doubts, sources of distress, and difficulties related to the treatment; principally at the time when they find out about the disease, as it is common to present irritability, guilt or apathy, among other negative feelings, a fact which can negatively influence these peoples' involvement for following the treatment^18^.

It is understood that the more that people living with AIDS are informed, the more capable they are for dealing with the situation, and the better they understand that changing their lifestyle and being committed to the treatment are crucial for a better prognosis of the disease, and, consequently, for a good quality of life. 

Participation in the treatment can be significantly linked to the amount of information which the individuals have regarding the disease, above all regarding the consequences which irregularity in the treatment can cause, such as opportunistic infections and systemic changes. 

The nurse can intervene in order to minimize the predictive factors for the nursing diagnosis of knowledge deficit, through guidance regarding the disease, the prognosis, treatment and living habits which can favor the improvement of quality of life. It is also necessary to take into account how this information will be passed on, principally to those with a low educational level. 

In relation to the predictive factor of years of study, linked to the nursing diagnosis of failure to adhere, studies indicate that the low educational level of people living with AIDS predominates as an aspect which can influence lack of adherence to the treatment of the disease^3^ ^-^ ^4^ ^,^ ^19^.

In one study undertaken in the United States of America, which aimed to analyze the relationship between the individuals' knowledge regarding HIV and their adherence to the treatment, it was ascertained that knowledge on their state of health, including aspects related to the disease's development, such as CD4 count and viral load, contribute to the involvement of people living with AIDS in their adherence to the treatment^17^.

Other factors are indicated by some studies as possibly influencing adherence to the treatment: financial difficulties, nutritional factors, lack of belief in the treatment, drug use, depressive symptoms, the occurrence of adverse reactions during the taking of medications, and the quantity of antiretrovirals ingested per day^16^ ^,^ ^20^ ^-^ ^21^.

The financial difficulties limit access to appropriate food for minimizing the situation of physical debility, characterized by muscular weakness and low weight, resulting from the action of the virus itself, as well as the adverse reactions to the medications, or symptoms of the opportunistic infections^20^ ^-^ ^22^. Lack of belief in the treatment, on the other hand, may be related to an understanding of the inefficacy of the antiretrovirals, due to the fact that this is an incurable disease^18^.

Besides this, adverse events resulting from these medications can also involve anatomical, metabolic or neuropsychiatric changes, as well as gastrointestinal effects of different intensities^12^.Another important aspect relates to the quantity of antiretrovirals ingested per day, which also contributes to people living with AIDS becoming resistant to taking medication or not recording the times that they take the tablets^12^ ^,^ ^19^.

It is known, however, that the antiretroviral medications have an important role in the control, survival, and improvement of quality of life of people living with AIDS, as they act in viral suppression and maintain the levels of the CD4 cells at a higher level, thus allowing an improvement in the immunological system and a reduction in the occurrence of opportunistic infections^13^ ^,^ ^15^.

It is important to emphasize that, although in this study there was no statistical significance between the diagnosis of failure to adhere and the variables related to the consumption of alcoholic drinks, tobacco and illicit drugs, it is interesting to note that the factors related to behavior which is indicative of lack of adherence and forgetfulness can result from this lifestyle, which is highly prevalent among the participants in this research.

Studies confirm that unhealthy life behaviors, including the consumption of drugs, can be determined both in contagion with HIV and in relation to adherence to the disease's treatment, as these people commonly - when ingesting alcohol or consuming other drugs - reduce their level of cognition and undertake specified behaviors, such as the practice of unsafe sex, multiplicity of partners, the sharing of syringes, and forgetting to take their antiretroviral medications^20^ ^-^ ^23^. Furthermore, the drug users, due to their high degree of dependency, prefer to take drugs rather than ingest the medications, bearing in mind that the concomitant use can increase the adverse effects of the antiretrovirals, or even because of the fact that they consider the use of drugs to be mechanisms of escape from life's adversities^12^ ^,^ ^21^.

In this regard, the nurse must be alert to the multiple factors which influence adherence to the treatment, principally those referent to the behavioral questions. Involvement and the feeling of responsibility are fundamental for therapeutic success. Family and psychological support are also necessary in this process, in the sense of encouraging people living with AIDS to follow the treatment appropriately, including those behavioral changes which have such an impact for a better prognosis of the disease. It is necessary to involve these people in their health/illness process, principally by providing them with the possibility to choose, in conjunction with the health team, how the treatment will be carried out, also taking into account the extrinsic factors such as the socioeconomic and cultural factors. 

The nursing diagnosis of sexual dysfunction presented, as predictive factors, altered body function, family income, reduced frequency of sexual practice, perceived deficit in sexual desire and perceived limitations imposed by the disease. 

One study indicates that great discomfort exists in relation to the bodily changes caused by AIDS and the antiretrovirals, mainly those related to the loss of weight and to lipodystrophy. This change in body image sometimes can cause insecurity or shame in people living with AIDS, being able to negatively influence their self-esteem and their personal and sexual relationships, principally when this is related to a situation of depression^4^.

The principal form of transmission of the disease can negatively influence the way that people living with AIDS experience their sexuality, whether through fear of transmitting the virus, or through associating sexual practice with their current health condition, mainly when these people present various episodes of inpatient treatment in a short period of time^4^.

Sexual dysfunction has multifactorial causes, which can be organic or psychological. Academics indicate that endothelial dysfunction can occur as a consequence of a situation of dyslipidemia, obesity, or diabetes mellitus, caused by the action of HIV itself, or by the adverse effects of the antiretrovirals. This process changes the vascular relaxation and blood flow, thus negatively influencing the sexual functioning of people living with AIDS^24^.

Hypogonadism has multifactorial pathogenesis, and can occur because of the cytopathic effects of the virus in testicular cells, the use of gonadal toxic drugs, malnutrition, opportunistic infections and the neoplasias which affect the testicle. The low levels of testosterone can be related to hypogonadism and can cause loss of muscle mass and strength, reduction in bone mineral density, and lipodystrophy and sexual dysfunction^24^.

Finally, the nursing diagnosis of sexual dysfunction presented family income as one of its predictive factors. Living with a disease which continues to be so stigmatized by society hinders the individual's access to the labor market^4^, contributing to unemployment and, consequently, financial difficulties. 

In the light of the multi-causality which can involve the nursing diagnosis of sexual dysfunction, it is important for the nurse to establish a relationship of trust and respect for the people living with AIDS for whom she provides care, so that she may come to know the predictors which can lead to these dysfunctions occurring, or which have already caused an impact on these peoples' quality of life. 

As contributions to the advance of scientific knowledge, it is emphasized that the identification of predictive factors confers upon the nurse greater clinical power, as it allows the knowledge of those aspects which are related to the human responses of people living with AIDS, and the extent to which these possess a multicausal dimension and are interlinked. This identification facilitates the establishment of nursing interventions which are more suited to these peoples' real needs, and to their socioeconomic and cultural context, as well as making it possible to minimize the complications of the treatment and the coexistence with the disease itself; also being able to provide a basis for the teaching of the nursing diagnoses. 

The study's limits were related to the non-probabilistic type of sampling, which does not guarantee the sample's representativity. Another aspect identified consists of the fact that the clinical evaluation is a subjective process; in the light of this, the diagnostic process is subject to uncertainties. It is stressed that the small number of studies addressing the nursing diagnoses in people living with AIDS, and which used inferential statistics, led to a certain difficulty for comparing the findings of the present study. 

In this regard, it is suggested that further studies should be undertaken with a view to giving a broader foundation for the nurse's care practice with people living with AIDS, principally in relation to the nursing diagnoses and their predictors. 

## Conclusion

The study made it possible to identify that the predictive factors of the nursing diagnoses of knowledge deficit, failure to adhere and sexual dysfunction, found in the people living with AIDS and involved sociodemographic and clinical characteristics, defining characteristics, and related factors, which can be used by nurses for identifying the increased risk for the development of specific human responses and complications related to the disease.

## References

[1] Granich R, Gupta S, Hersh B, Williams B, Montaner J, Young B (2015). Trends in AIDS Deaths, New Infections and ART Coverage in the Top 30
Countries with the Highest AIDS Mortality Burden; 1990-2013. PLoSOne.

[2] Faria JO, Silva GA (2013). Diagnósticos de enfermagem em pessoas com HIV/aids abordagem baseada
no modelo conceitual de Horta. Rev RENE.

[3] Faria JO, Silva GA. (2014). Diagnósticos de enfermagem do domínio segurança e proteção em pessoas
com HIV/Aids. Rev Eletrônica Enferm.

[4] Cunha GH, Galvão MTG (2010). Diagnósticos de enfermagem em pacientes com o Vírus da
Imunodeficiência Humana/ Síndrome da Imunodeficiência Adquirida em assistência
ambulatorial. Acta Paul Enferm.

[5] Gómez JJ, Mayorga CME, Pérez MJO, Rojas SLZ, Orozco VLC, Camargo FFA (2013). Prevalencia de diagnósticos de enfermería en personas con
VIH/SIDA. Enferm Glob.

[6] Brasileiro ME, Cunha LC. (2011). Diagnósticos de enfermagem em pessoas acometidas pela Síndrome da
Imunodeficiência Adquirida em terapia antirretroviral. Rev Enferm UERJ.

[7] Fontelles MJ, Simões MG, Almeida JC, Fontelles RG. (2010). Metodologia da pesquisa: diretrizes para o cálculo do tamanho da
amostra. Rev Paran Med..

[8] Lira ALBC, Lopes MVO (2010). Pacientes transplantados renais análise de associação dos diagnósticos
de enfermagem. Rev Gaúcha Enferm.

[9] Herdman TH (2012). International nursing diagnoses: definitions and classification,
2012-2014..

[10] Souza CC, Mata LRF, Azevedo C, Gomes CRG, Cruz GECP, Toffano SEM (2013). Interiorização do HIV/aids no Brasil um estudo
epidemiológico. Rev Bras Ciênc Saúde.

[11] Kotwal J, Singh V, Kotwal A, Dutta V, Nair V (2013). A study of haematological and bone marrow changes in symptomatic
patients with human immune deficiency virus infection with special mention of
functional iron deficiency, anaemia of critically ill and
haemophagocyticlymphohistiocytosis. Med J Armed Forces India.

[12] Sousa MP, Luna IT, Silva KL, Pinheiro PNC (2012). Pacientes vivendo com HIV/aids e coinfecção tuberculose dificuldades
associadas à adesão ou ao abandono do tratamento. Rev Gaúcha Enferm.

[13] Moges NA, Kassa GM (2014). Prevalence of opportunistic infections and associated factors among
HIV positive patients taking anti-retroviral therapy in Debre Markos referral
hospital, northwest Ethiopia. J AIDS Clin Res.

[14] Santos AC, Almeida AM (2013). Nutritional Status and CD4 Cell Counts in Patients with HIV/AIDS
Receiving Antiretroviral Therapy. Rev Soc Bras Med Trop.

[15] Mitiku H, Weldegebreal F, Teklemariam Z (2015). Magnitude of opportunistic infections and associated factors in
HIV-infected adults on antiretroviral therapy in eastern Ethiopia. HIV/AIDS - Res Palliative Care.

[16] Saharia KK, Koup RA (2013). T cell susceptibility to HIV influences outcome of opportunistic
infections. Cell.

[17] Jones D, Cook R, Rodriguez A, Valverde DW (2013). Personal HIV Knowledge, Appointment Adherence and HIV
Outcomes. AIDS Behav.

[18] Camargo LA, Capitão CG, Filipe EMV (2014). Mental health, family support and treatment adherence associations in
the context of HIV/aids. Psico USF.

[19] Melo GC, Rodrigues STC, Trindade RFC, Holanda JBL. (2014). Adesão ao tratamento: representações sociais sobre a terapia
antirretroviral para pessoas que vivem com HIV. Rev Enferm UFPE.

[20] Berhe N, Tegabu D, Alemayehu M (2013). Effect of nutritional factors on adherence to antiretroviral therapy
among HIV-infected adults a case control study in Northern
Ethiopia. Infectious Dis.

[21] Barroso J, Voss JG (2013). Fatigue in HIV and aids An Analysis of Evidence. J Assoc Nurses Aids Care.

[22] Fagbami O, Oluwasanjo A, Fitzpatrick C, Fairchild R, Shin A, Donato A (2015). Factors Supporting and Inhibiting Adherence to HIV Medication Regimen
in Women A Qualitative Analysis of Patient Interviews. Open AIDS J.

[23] Naidoo P, Chirinda W, Mchunu G, Swartz S, Anderson J (2014). Social and structural factors associated with vulnerability to HIV
infection among young adults in South Africa. Psychol Health Med.

[24] Romero-Velez G, Lisker-Cervantes A, Villeda-Sandoval CI, Zavaleta MS, Olvera-Posada D, Sierra-Madero JG, Arreguin-Camacho LO, Castillejos-Molina RA (2014). Erectile Dysfunction Among HIV Patients Undergoing Highly Active
Antiretroviral Therapy Dyslipidemia as a Main Risk Factor. Sex Med.

